# A thrombus detachment from the right coronary artery ostium caused cerebral infarction after coronary artery angiography: A case report

**DOI:** 10.1097/MD.0000000000045260

**Published:** 2025-10-17

**Authors:** Lingxiao Fang

**Affiliations:** a Department of Cardiology, Suining Central Hospital, Suining, Sichuan Province, China.

**Keywords:** thrombus detachment, the right coronary artery ostium, cerebral infarction, coronary artery angiography

## Abstract

**Rationale:**

Although coronary angiography (CAG) can occasionally lead to cerebral infarction, the detachment of a thrombus from the coronary ostium resulting in blockage of head and neck vessels is rare. We present a case of cerebral infarction caused by thrombus detachment from the right coronary artery (RCA) ostium following CAG. This case underscores the importance of accurate identification and management of coronary ostial thrombus during CAG.

**Patient concerns:**

A 72-year-old woman was admitted to the hospital with chest pain lasting for 2 hours.

**Diagnoses:**

Based on the electrocardiogram findings, the patient was diagnosed with an acute inferior ST-Segment Elevation Myocardial infarction accompanied by third-degree atrioventricular block in accordance with established guidelines.

**Interventions:**

The patient was promptly scheduled for CAG. During RCA angiography, the angiography catheter was inserted into the base of the aortic sinus, and a small volume of contrast agent was injected. The ostium of the RCA was observed to become opacified. However, after the catheter was engaged in the RCA ostium, no further opacification was observed upon contrast injection, and no stenosis was detected in the proximal, middle, or distal segments. We suspected that the thrombus had dislodged.

**Outcomes:**

The patient’s chest symptoms resolved immediately. Moreover, her mental status improved significantly, with normal motor strength and muscle tone in all 4 extremities. However, subsequent computed tomography angiography of the head and neck, cranial magnetic resonance imaging, and magnetic resonance angiography examinations revealed the presence of carotid artery thrombus and acute cerebral infarction.

**Lessons:**

This case underscores the critical importance of accurate identification and management of coronary ostial thrombus during CAG. Notably, when coronary ostial thrombosis is encountered, prompt postprocedural evaluation, including computed tomography angiography of the head and neck to assess for systemic embolization, is warranted.

## 1. Introduction

Although coronary angiography (CAG) can occasionally lead to cerebral infarction, thrombus detachment from the coronary ostium resulting in blockage of head and neck vessels is rare. A 72-year-old woman diagnosed with ST-Elevation Myocardial infarction underwent emergency CAG. During the procedure, the right coronary artery (RCA) ostium appeared opacified (“white”), raising suspicion of thrombus. However, after contrast injection, the opacification resolved, and no stenosis was observed in any coronary segments. Post procedurally, head and neck computed tomography angiography (CTA) revealed a thrombus in the carotid artery.

## 2. Case report

**Figure 1.**
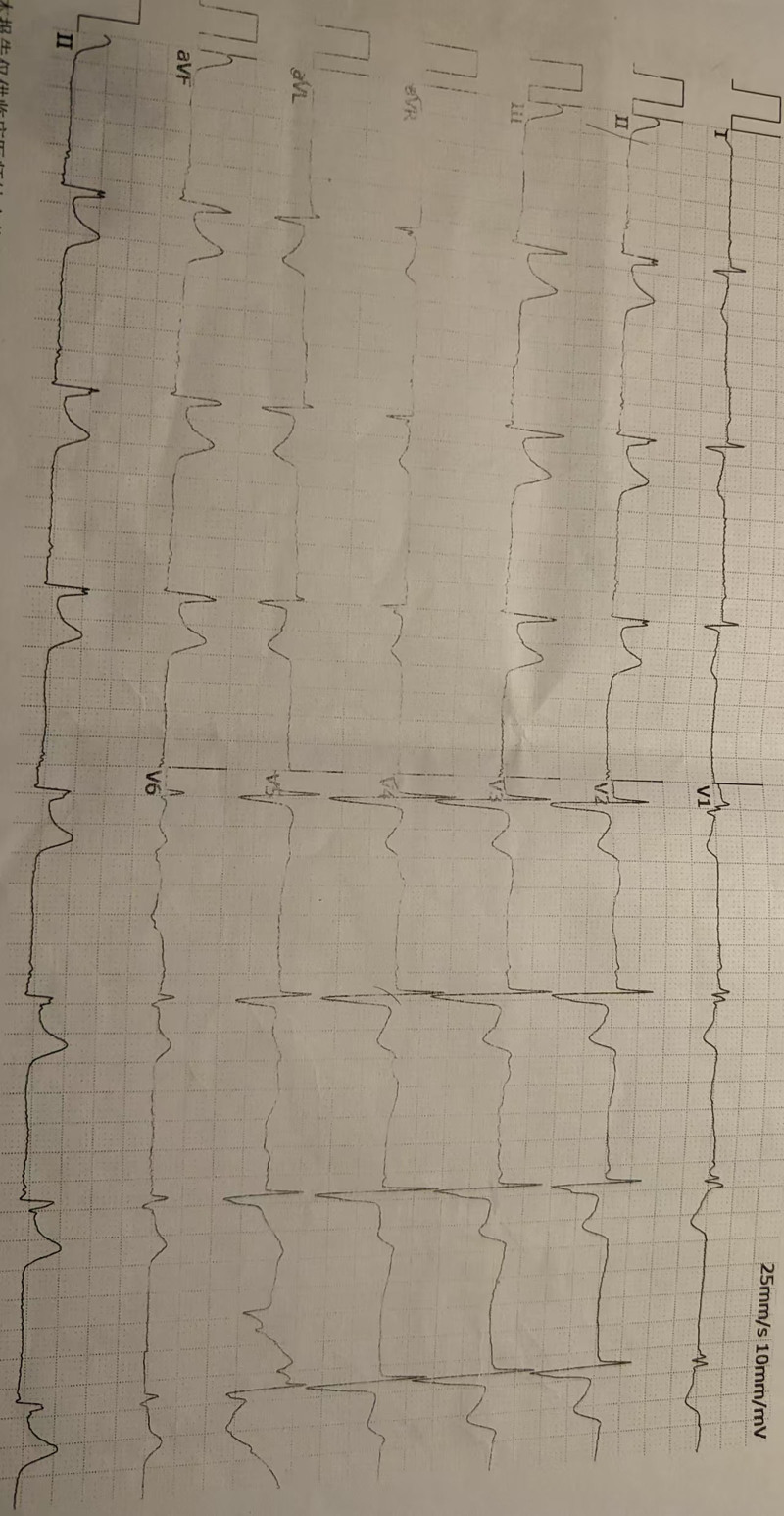
Baseline rhythm strips.

A 72-year-old woman was admitted to the hospital with chest pain lasting for 2 hours. The patient had no history of cardiovascular risk factors (such as hypertension, diabetes mellitus, or dyslipidemia), thromboembolic events, or use of antiplatelet/anticoagulant agents. Based on the electrocardiogram findings (Fig. 1), she was diagnosed with an acute inferior ST-Segment Elevation myocardial infarction accompanied by third-degree atrioventricular block in accordance with established guidelines.^[1]^ Consequently, she was promptly scheduled for CAG.

**Figure 2.**
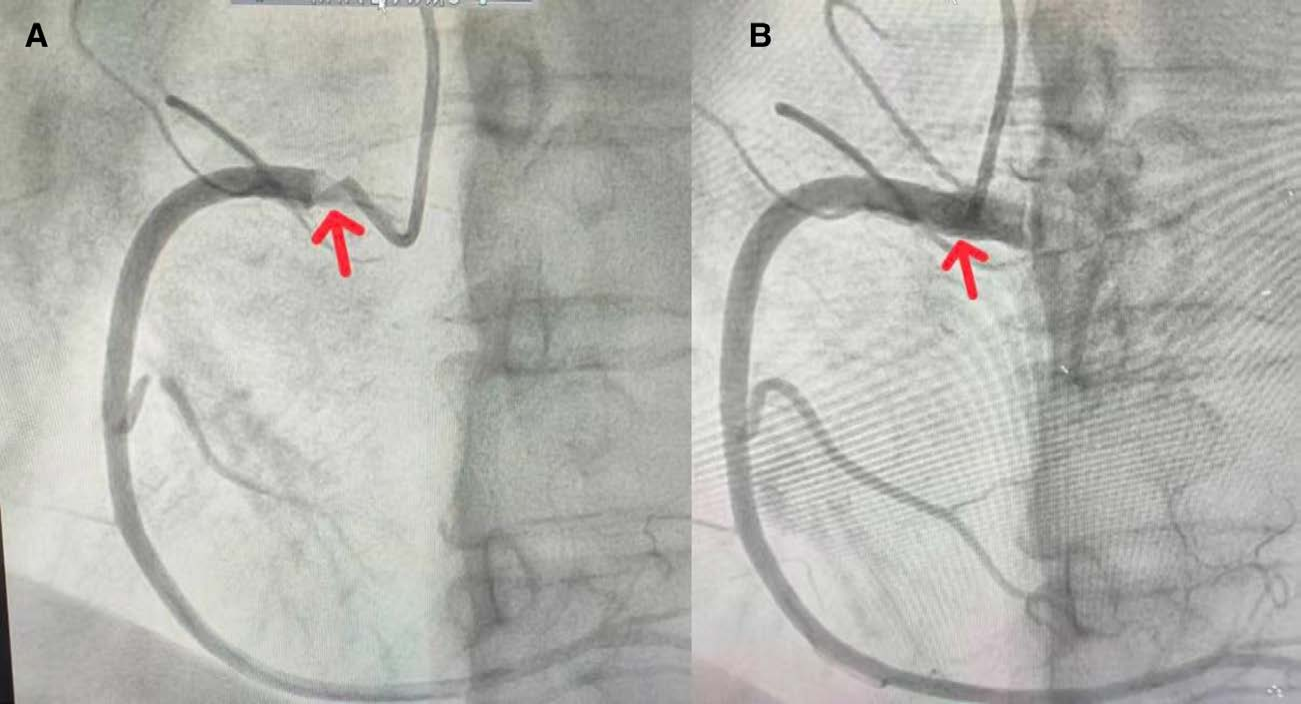
(A) Prior to CAG, the RCA ostium was observed to become opacified (appearing white). The location indicated by the red arrow corresponds to the RCA ostium. (B) After the catheter engaged the RCA ostium, no opacification was observed following contrast injection, and no stenosis was visible in the proximal, middle, or distal segments. The location indicated by the red arrow corresponds to the RCA ostium. CAG = coronary angiography, RCA = right coronary artery.

During RCA angiography, the catheter was inserted into the base of the aortic sinus, followed by injection of a small volume of contrast agent. The RCA ostium was observed to become opacified (appearing white). However, after the catheter engaged the RCA ostium, no opacification was observed following contrast injection, and no stenosis was visible in the proximal, middle, or distal segments (Fig. 2A and B). We suspected that the thrombus had dislodged. Concurrently, the patient’s chest symptoms resolved immediately.

After the procedure, the patient’s mental status improved significantly, and motor strength and muscle tone were normal in all 4 extremities. Transthoracic echocardiography was performed and revealed the following:

• Regional wall motion abnormalities with hypokinesis of the interventricular septum and multiple left ventricular segments.
• Mild regurgitation of both the mitral and aortic valves.
• Reduced left ventricular systolic function, with left ventricular ejection fraction estimated at 45% using Simpson’s biplane method.

**Figure 3.**
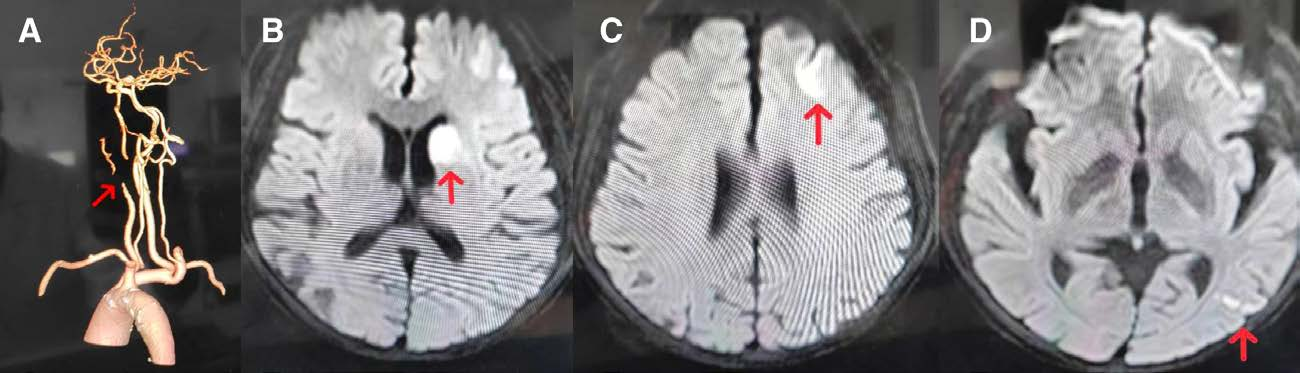
(A) Head and neck CTA demonstrated stenosis in the C1 segment of the left internal carotid artery, with the site of narrowing indicated by the red arrow. (B–D) Cranial MRI showing acute cerebral infarcts in the bilateral frontal lobes, left temporo-parieto-occipital region, and basal ganglia. The red arrow indicates the site of corresponding vascular stenosis. CTA = computed tomography angiography, MRI = magnetic resonance imaging.

To further identify the target organ of the thrombus had embolism, we performed a comprehensive postoperative evaluation using the head and neck CTA, cranial magnetic resonance imaging (MRI), and magnetic resonance angiography (MRA). Head and neck CTA revealed significant narrowing of the left common carotid artery, the C1 segment of the internal carotid artery, and the left external carotid artery, with low-density intraluminal shadows suggestive of thrombus. Severe stenosis and local occlusion were observed (Fig. 3A). Prio to CAG, the patient had not received any anticoagulant. Upon identification of a thrombus in the common carotid artery by CTA, anticoagulation therapy with rivaroxaban (15 mg once daily) was immediately initiated. Cranial MRA confirmed the presence of thrombus in the left common carotid artery, the C1 segment of the internal carotid artery, and the origin of the external carotid artery, accompanied by severe stenosis and luminal occlusion. Cranial MRI revealed cerebral infarctions in the bilateral frontal lobes, the left temporo-parieto-occipital lobe, and the basal ganglia region, consistent with the acute phase of the condition (Fig. 3B–D).

Although further cerebral angiography was recommended, the patient declined the procedure due to financial constraints and requested discharge. Arrangements were subsequently made for the patient’s discharge.

The sequence of clinical events is presented by a bullet format below:

• 2 March: → 13:39: The patient sought medical attention for acute chest pain.
  → 13:50: The patient was diagnosed with STEMI based on ST-segment elevation.
  → 14:50: Interventional cardiologists performed emergent CAG via radial access.
  → 15:00: The patient reported resolution of chest pain following CAG.
• 4 March: → 11:00: The patient underwent urgent head and neck CTA.
• 5 March: → 10:45: The patient underwent cranial MRI and MRA.

## 3. Discussion

The occurrence of cerebral infarction in this patient following emergency CAG was entirely unanticipated. Moreover, the patient exhibited no symptoms or signs suggestive of cerebral infarction during the postoperative period.

The presence of sinus rhythm effectively excluded atrial fibrillation as an embolic source for simultaneous cerebral and myocardial infarction. Unfortunately, transesophageal echocardiography was not performed to assess for potential intracardiac thrombus. We postulated that the thrombus was located precisely at the ostium of the RCA. During contrast injection into the RCA, hemodynamic forces may have caused thrombus dislodgement into the aorta. Subsequently, the embolus traveled retrogradely and entered the cerebral circulation, leading to vessel occlusion. Regrettably, no further imaging was performed to evaluate whether the dislodged thrombus had also caused obstruction in other vascular beds, such as the renal or superior mesenteric arteries.

This case underscores the critical importance of accurate identification and management of coronary ostial thrombus during CAG. It is noteworthy that when coronary ostial thrombosis is encountered, prompt postoperative assessment, including head and neck CTA, should be conducted to evaluate potential systemic embolization.

### 3.1. Limitations

First, cerebral angiography was not performed to evaluate for cerebrovascular disease. Second, transesophageal echocardiography was not conducted to further assess for potential thrombus. Third, contrast-enhanced abdominal CT was not obtained to evaluate for mesenteric artery embolism, among other possibilities.

## Author contributions

**Formal analysis:** Lingxiao Fang.

**Investigation:** Lingxiao Fang.

**Supervision:** Lingxiao Fang.

**Writing – original draft:** Lingxiao Fang.

**Writing – review & editing:** Lingxiao Fang.
